# Aryl-4,5-dihydro-1*H*-pyrazole-1-carboxamide Derivatives Bearing a Sulfonamide Moiety Show Single-digit Nanomolar-to-Subnanomolar Inhibition Constants against the Tumor-associated Human Carbonic Anhydrases IX and XII

**DOI:** 10.3390/ijms21072621

**Published:** 2020-04-09

**Authors:** Priya Hargunani, Nikhil Tadge, Mariangela Ceruso, Janis Leitans, Andris Kazaks, Kaspars Tars, Paola Gratteri, Claudiu T. Supuran, Alessio Nocentini, Mrunmayee P. Toraskar

**Affiliations:** 1 Bharati Vidyapeeth’s College of Pharmacy, Navi Mumbai 400 614, India; phargunani@yahoo.in (P.H.); niktadge@gmail.com (N.T.); 2 Neurofarba Dept., Section of Pharmaceutical and Nutraceutical Sciences, Universitá degli Studi di Firenze, Via U. Schiff 6, Sesto Fiorentino, 50019 Florence, Italy; mariangela.ceruso@unifi.it (M.C.); paola.grateri@unifi.it (P.G.); claudiu.supuran@unifi.it (C.T.S.); 3 Latvian Biomedical Research and Study Center, Ratsupites 1, LV-1067 Riga, Latvia; janis.leitans@biomed.lu.lv (J.L.); andris@biomed.lu.lv (A.K.); kaspars@biomed.lu.lv (K.T.)

**Keywords:** sulfonamide, pyrazoline, human carbonic anhydrase, inhibition, selectivity, docking

## Abstract

A series of new 3-phenyl-5-aryl-*N*-(4-sulfamoylphenyl)-4,5-dihydro-1*H*-pyrazole-1-carboxamide derivatives was designed here, synthesized, and studied for carbonic anhydrase (CAs, EC 4.2.1.1) inhibitory activity against the human (h) isozymes I, II, and VII (cytosolic, off-target isoforms), and IX and XII (anticancer drug targets). Generally, CA I was not effectively inhibited, whereas effective inhibitors were identified against both CAs II (K_I_s in the range of 5.2–233 nM) and VII (K_I_s in the range of 2.3–350 nM). Nonetheless, CAs IX and XII were the most susceptible isoforms to this class of inhibitors. In particular, compounds bearing an unsubstituted phenyl ring at the pyrazoline 3 position showed 1.3–1.5 nM K_I_s against CA IX. In contrast, a subset of derivatives having a 4-halo-phenyl at the same position of the aromatic scaffold even reached subnanomolar K_I_s against CA XII (0.62–0.99 nM). Docking studies with CA IX and XII were used to shed light on the derivative binding mode driving the preferential inhibition of the tumor-associated CAs. The identified potent and selective CA IX/XII inhibitors are of interest as leads for the development of new anticancer strategies.

## 1. Introduction

Carbon dioxide (CO_2_) plays a vital role in all life processes. It exists in equilibrium with bicarbonate, but the interconversion is a slow process [1]. Carbonic anhydrases (CAs, EC 4.2.1.1) are metalloenzymes that catalyze this reversible hydration reaction of carbon dioxide to bicarbonate and protons [1,2]. As a result, CAs play an important role in respiration, pH and CO_2_ homeostasis, electrolyte secretion in a variety of tissues and organs, biosynthetic reactions (such as gluconeogenesis and lipid and urea synthesis), bone resorption, tumorigenicity, and many other physiological or pathological processes [3]. Fifteen different α-carbonic anhydrase isoforms have been isolated and characterized in humans and many of them are well-established therapeutic targets to treat a wide range of disorders [4]. Based on cellular and sub-cellular location, CAs are classified into four different groups: Cytosolic (CA I, II, III, VII, XIII), mitochondrial (CA VA, VB), secretory (CA VI), and membrane-associated (CA IV, IX, XII, XIV, XV) [5]. The active site of CA is a deep cleft, on one side of which only hydrophobic residues are present, whereas hydrophilic residues line the opposite side. The Zn^2+^ ion is coordinated tetrahedrally by three imidazoles of conserved histidine residues (His94, His96 and His119), and a water molecule/hydroxide ion is a fourth ligand [2]. The membrane-bound CA IX and XII isoforms are known as the CAs associated with cancers, being expressed in a limited number of normal tissues [5,6,7,8,9]. Solid tumors usually have a dynamic microenvironment characterized by acidic pH and low levels of oxygen (i.e., hypoxia). For survival, cancer cells acquire different adaptive features, which are most probably responsible for the development of invasive and metastatic phenotypes. As a response to regional hypoxia, cancer cells obtain their need for high levels of ATP synthesis by switching their metabolism from aerobic respiration to fermentative (anaerobic) glycolysis [10]. CA IX and XII regulate tumor cell pH by maintaining an intracellular slightly alkaline pH (of 7.2–7.4) while acidifying the extracellular pH (which arrives at 6.0–6.5), and this is exploited by the cancer cells for survival and proliferation [6]. The hypoxic conditions in solid tumors induce overexpression of CA IX and CA XII, which maintain the acidic tumor environment by catalyzing the reversible hydration of tumor cell-generated CO_2_ into a bicarbonate anion (HCO_3_^−^) and a proton (H^+^) and then trap H^+^ extracellularly to lower the pH [5,6]. Hence, the overexpression of these isoforms contributes to the increased acidification of the extracellular hypoxic environment in contrast to normal tissues. Expression of both membrane-associated CAs is induced by hypoxia in breast tumors and in several cancer cell lines [7,8]. CA XII expression in breast tumors is indicative of lower grade disease, lower relapse rates, and better overall patient survival [9]. In contrast, CA expression in brain tumors is associated with poor prognosis [9,10].

Although inhibition of hCAs by aromatic and heterocyclic sulfonamides has been largely studied for 50 years with applications in the treatment of various disorders [2,4], these classes of CA inhibitors (CAIs) remain an attractive chemical family in the design of new inhibitors, as shown by the ongoing research in this field [11,12,13]. The primary sulfonamide group binds to the Zn^2+^ ion in the CA enzyme active site as an anion (SO_2_NH^−^) blocking the catalysis [11]. A wealth of recent studies has shown that the presence of urea as a linker in sulfonamide-containing compounds is beneficial to generating effective CA inhibitory activity [14,15]. In the present study, novel 3-phenyl-5-aryl-*N*-(4-sulfamoylphenyl)-4,5-dihydro-1*H*-pyrazole-1-carboxamide derivatives were designed and synthesized on the basis of sulfonyl semicarbazides and iminoureido CAIs previously synthesized in our laboratory [16,17]. In vitro inhibition studies were carried out for all newly synthesized compounds against CA I, II, VII, IX, and XII. The synthesized derivatives were thoroughly characterized by ^1^H-NMR, ^13^C-NMR, IR, and MS, and their purity was checked by HPLC.

## 2. Results and Discussion

### 2.1. Chemistry

**Scheme 1:**
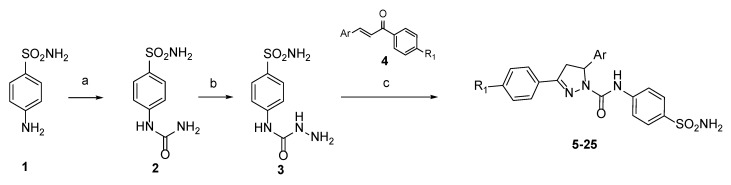
Synthesis of compounds **5**–**25**. Reagents and conditions: a. NaCNO, glacial AcOH; b. NH_2_NH_2_.H_2_O, EtOH, reflux, 27 h; c. EtOH, KOH, reflux, 2–3 h, overnight stirring.

In the present work, a new series of 3-phenyl-5-aryl-*N*-(4-sulfamoylphenyl)-4,5-dihydro-1*H*-pyrazole-1-carboxamide (**5**–**25**) is reported. Initially, 4-ureidobenzenesulfonamide **2** was obtained from reaction of sulfanilamide with sodium cyanate in ethanol and water, which was further refluxed with hydrazine hydrate in ethanol to obtain 4-aminosulphonylphenyl semicarbazide **3**. Various chalcones of the general formula **4** were prepared by Claisen–Schmidt condensation of the appropriate 4-chloro/bromophenyl acetophenone with substituted aromatic/heteroaromatic aldehydes in the presence of NaOH. Cyclization of semicarbazide **3** with chalcones in the presence of a base afforded the corresponding 3-phenyl-5-aryl-*N*-(4-sulfamoylphenyl)-4,5-dihydro-1*H*-pyrazole-1-carboxamide (**5**–**25**) (Scheme 1), accomplishing the chemical hybridization of sulfanilamide with pyrazoline. The chemical structures of titled compounds **5**–**25** were established on the basis of spectroscopic analysis. In detail, compounds **5**–**25** were characterized by the presence of the three prominent doublets of doublets, each having the integration of one proton. Formation of the pyrazoline was confirmed by the presence of a doublet of doublets for CH_2_ at C_4_ giving a signal at δ 3.26–3.13 and δ 3.93–3.83 ppm, and CH at C_5_ giving a signal at δ 5.54–5.48 ppm. The general procedures for the synthesis and spectral data are described in the Experimental Section.

### 2.2. Carbonic Anhydrases Inhibition

Sulfonamides **5**–**25** were evaluated for their inhibition against the cytosolic CA I, II, and VII and the membrane-bound CA IX and XII by using a stopped-flow CO_2_ hydrase assay method [18]. The clinically used acetazolamide (AAZ) was used as standard drug in the kinetic evaluation. The following SAR can be worked out from the data reported in Table 1.

CA I was the least inhibited isoform by compounds **5–25** with inhibition constants (K_I_s) ranging between 36.2 and 769.6 nM. The 4-Br substitution as R (23–25) stood out as the most efficient for providing <100 nM K_I_s (i.e., 38.2–45.5 nM), though a comparison can be made on a limited set of Ar substituents. By contrast, when analog Ar groups are considered, a H atom (5–11) produced better CA I inhibition than a chlorine atom (12–22).

The most physiologically relevant isoform, CA II, was efficiently inhibited by most derivates with K_I_s nearby or even below 10 nM (5.2–13.5 nM), except for 5, 7, 12, and 15. The phenyl derivative 12 induced significantly worse CA II inhibition than most of its relative compounds (K_I_ of 233 nM).

The last tested cytosolic CA, isoform VII, was even more affected than CA II, with the K_I_s compacting in a very narrow range spanning between 2.3 and 7.7 nM, comparably to the standard AAZ (K_I_ of 2.5 nM). Compounds 5 and 17 (K_I_s of 33.2 and 27.6 nM) reported a 10-fold worse CA VII inhibition, whereas a drop in efficacy was measured for the 4-Cl derivative 12 (K_I_ of 350 nM), which also exhibited a reduced action against CA II when compared to the other derivatives. 

Undoubtedly, the activity of the tumor-associated CAs IX and XII was the most susceptible to inhibition by the sulfonamides **5–25** reported here. Most derivatives showed an extremely narrow range of inhibition of CA IX as K_I_s spanned in the range 1.3–2.9 nM. Again, compound 12 together with 14 and 24 exhibited slightly increased K_I_s (11.4–16.5 nM), whereas the 2-hydroxy derivative 20 turned out as the less active CA IX inhibitor in the study. It should be noted that compounds bearing an unsubstituted phenyl ring at the pyrazoline 3 position showed the best K_I_s against CA IX (1.3–1.5 nM).

Even subnanomolar inhibition was achieved against CA XII by some screened compounds (K_I_s of 0.62–0.99 nM), where another number oddly showed K_I_s settling around 1 nM. It is interesting to note that subnanomolar inhibitions are achieved uniquely by derivatives having a 4-halo-phenyl at the 3 position of the aromatic scaffold. The less active inhibitors 12 and 13 exhibited a K_I_ of 7.7 nM. Further discussion on the SAR cannot be produced, because of the extremely narrow trend of inhibition measured for **5–25** against all CAs.

Derivatives 5–25 act as selective inhibitors against the tumor-associated CAs IX and XII over CA II and mostly CA I, with a selectivity index even reaching four-digit values (e.g., CA I/XII SI of compound 18).

### 2.3. Molecular Docking

**Figure 1:**
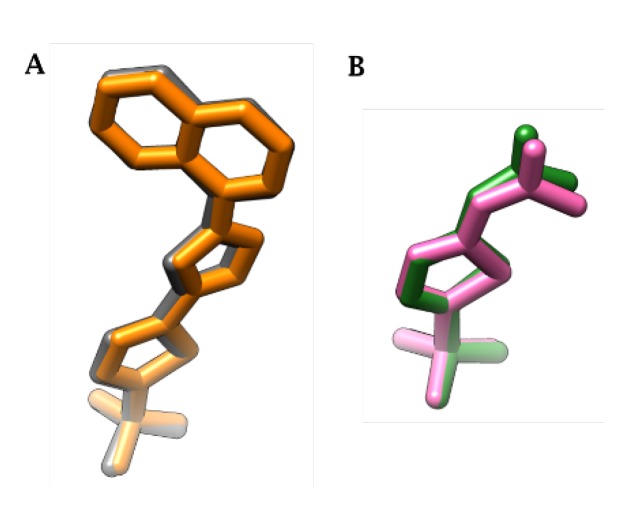
Superimposition of docked (orange and pink) and crystallographic (grey and green) poses of (**A**) 5-(1-(naphthalen-1-yl)-1H-1,2,3-triazol-4-yl)thiophene-2-sulfonamide bound to CA IX in pdb 5FL4, and (**B**) **AAZ** bound to CA XII in pdb 1JD0.

The binding mode of sulfonamides **5**–**25** to CA IX (pdb 5FL4) [19] and XII (pdb 1JD0) [8], the most susceptible isoforms to this class of CAIs, was predicted by docking. The effectiveness of the adopted computational procedure was testified by a root-mean-square deviation (RMSD) of only 0.49 and 0.35 Å between docked and crystallographic poses of the ligands present in pdb 5FL4 and pdb 1JD0, respectively (Figure 1).

**Figure 2:**
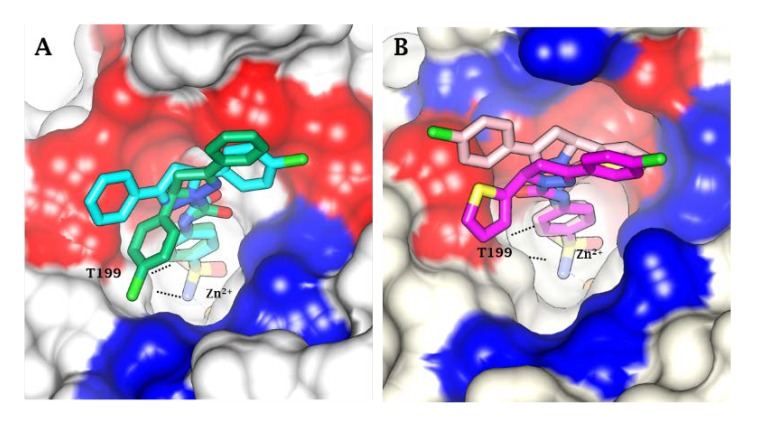
Surface representation of (**A**) CA IX (pdb 5FL4) bound to ***(R)*-6** (green) and ***(S)*-6** (cyan) and (**B**) CA XII (pdb 1JD0) bound to ***(R)*-18** (magenta) and ***(S)*-18** (pink). Lipophilic and hydrophilic residues are colored red and blue, respectively.

The in silico simulation was extended to both enantiomers ***(R)*** and ***(S)*** for each ligand. Coordination of the Zn ion from both isozymes occurs by the SO_2_NH^−^ group of all ligands, which is further involved in two H-bonds with the OH group and backbone NH of T199 (Figure 2). The aromatic core bearing the SO_2_NH_2_ forms π-alkyl contacts with L198, H94, and V121. 

**Figure 3:**
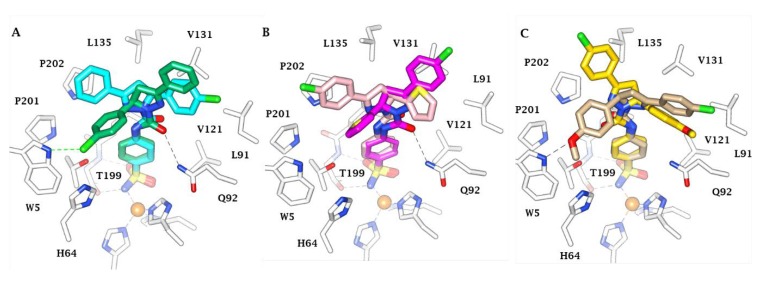
Docked binding orientations of (**A**) ***(R)*-6** (green) and ***(S)*-6** (cyan), (**B**) ***(R)*-18** (magenta) and ***(S)*-18** (pink), and (**C**) ***(R)*-21** (tan) and ***(S)*-21** (gold) to CA IX (pdb 5FL4). H-bonds are represented as dashed lines.

**Figure 4:**
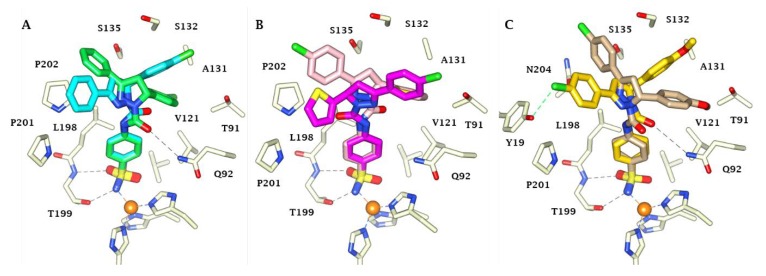
Docked binding orientations of (**A**) ***(R)*-6** (green) and ***(S)*-6** (cyan), (**B**) ***(R)*-18** (magenta) and ***(S)*-18** (pink), and (**C**) ***(R)*-21** (tan) and ***(S)*-21** (gold) to CA XII (pdb 1JD0). H-bonds are represented as dashed lines.

Figure 3 and Figure 4 depict the detailed interaction mode of both enantiomers of compound **6** (panel A), **18** (panel B), and **21** (panel C) superimposed within the active site of CA IX and XII, respectively. In CA IX, the orientations of the phenyl and aryl groups respectively in position 3 and 5 of the pyrazoline scaffold almost swap from the ***(R)***- to ***(S)***-enantiomer, and vice versa (Figure 3). In fact, the 3-phenyl portion in the ***(R)***-enantiomer and the 5-aryl moiety in the ***(S)***-enantiomer accommodate in the cleft region lined by V131, L91, and Q92. Contrariwise, the 3-phenyl portion in the ***(S)***-enantiomer and the 5-aryl moiety in the ***(R)***-enantiomer orient toward the edge between the hydrophilic and lipophilic regions of CA IX binding clefts (P201, P202, H64, and W5).

Similarly, an almost opposite binding orientation was found for the molecular tail of the ***(R)***- and ***(S)***-enantiomers of derivative **18** when bound to CA XII (Figure 4B). By contrast, by a 120° rotation of the pyrazoline orientation, the 3-phenyl groups from the two enantiomers of compounds **6** and **21**, as well as their 5-aryl groups, occupy the same area within the active site of CA XII (Figure 4A,C). It can be speculated that the markedly less lipophilic character of the “hydrophobic region” of the CA XII active site with respect to CA IX (Figure 2) attracts the most hydrophilic pendant between those at the pyrazoline position 3 and 5, in the case where a significant difference among them exists (e.g., compounds **6** and **21**).

It can also be noted that the ureido carbonyl group of the ***(R)***-enantiomers can accept a H-bond from Q92 side chain CONH_2_ within both CAs active sites (Figure 3 and Figure 4). 

The binding modes depicted in Figure 2, Figure 3 and Figure 4 are expected to be hindered within the CA II active site where the mutation of V/A131 to F131 significantly narrows the volume of the cavity. As a result, the CA II inhibition efficiency of **5**–**25** drops when compared to the tumor-related CAs, and so that against CA I does so because of the tight active site of such an isoform.

According to the docking scores computed for both optical isomers of compounds **5**–**25** reported in Table 2, the role of eutomers might be ascribed to *(S)*-enantiomers in the binding to CA IX (GlideScore –5.86–7.01 kcal/mol) and to *(R)*-enantiomers in the binding to CA XII (GlideScore –6.48–7.92 kcal/mol). No further comparison can be made between Glide-predicted scores and K_I_s determined in vitro. In fact, the GlideScore function is assumed to distinguish compounds that bind strongly from those that do not, and compounds **5**–**25** all act as low nanomolar CA IX and XII inhibitors (and even nanomolar CAIs against isoforms I and II). The docking scores computed for **AAZ** when bound within CA IX and XII active sites are indeed comparable to those of the eutomers of **5**–**25**.

## 3. Conclusions

A series of new 3-phenyl-5-aryl-*N*-(4-sulfamoylphenyl)-4,5-dihydro-1*H*-pyrazole-1-carboxamide derivatives was designed here, synthesized, and studied for hCA inhibitory activity against the cytosolic, off-target isoforms I, II, and VII and the anticancer drug targets IX and XII. While CA I was not effectively inhibited, effective inhibitors were identified against both CAs II (K_I_s in the range of 5.2–233 nM) and VII (K_I_s in the range of 2.3–350 nM). Nonetheless, CAs IX and XII were the most susceptible isoforms to this class of inhibitors. In particular, compounds bearing an unsubstituted phenyl ring at the pyrazoline 3 position showed 1.3–1.5 nM K_I_s against CA IX. By contrast, a subset of derivatives having a 4-halo-phenyl at the same position of the aromatic scaffold even reached subnanomolar K_I_s against CA XII (0.62–0.99 nM). Docking studies with CA IX and XII, performed on both enantiomers of the ligands, showed the binding mode driving such a preferential inhibition of the tumor-associated CAs. The identified potent and selective CA IX/XII inhibitors could represent interesting leads for the development of new anticancer medications.

## 4. Experimental Section

### 4.1. Chemistry

All the reagents and solvents were obtained from commercial suppliers and were used as received unless otherwise indicated. Solvents were dried, wherever necessary, according to standard procedures. All reactions were performed under N_2_ atmosphere, unless otherwise indicated. Analytical silica gel 60 F_254_-coated TLC plates were purchased from Sigma-Aldrich and were visualized with UV light. IR spectra (ATR) were recorded on a Quest ATR Diamond Accessory (Black) P31482 and Shimadzu 8100 infrared spectrophotometer. ^1^H-NMR was recorded at 300 MHz in DMSO-*d*_6_ solvents using TMS as an internal reference standard at Sophisticated Analytical Instrument Facility (SAIF) IIT Powai, Mumbai. Molecular ion peaks of synthesized compounds were recorded using HRLCMS at Laxai-Avanti Life Sciences Pvt. Ltd. Hyderabad. Melting points were recorded using a Veego^®^ (VMP)-D capillary melting point apparatus (Veego Instruments Corp. Mumbai, India) and were uncorrected.

#### 4.1.1. Synthesis of 4-ureidobenzenesulfonamide 2

In a 250 mL beaker, sulfanilamide (0.03 mol) was dissolved in a mixture of glacial HOAc (13 mL) and hot water (30 mL). A solution of sodium cyanate (0.05 moL) in hot water (25 mL) was added to the above mixture with continuous stirring. It was allowed to stand for 30 min. The reaction mixture was then cooled in an ice-bath, vacuum-filtered, dried, and recrystallized from EtOH. Yield: 70%; mp: 175 °C; IR (KBr) cm^−1^: 3462 (N–H *str* of NH_2_), 3365 (N–H *str*), 3227 (aromatic C–H *str*), 1693 (C=O *str*), 1533 (aromatic C=C *str*), 1409, 1321, 1155 (S=O *str*).

#### 4.1.2. Synthesis of 4-aminosulfonylphenyl Semicarbazide 3

In a 250 mL round bottom flask, equimolar quantities of **1** (0.05 mol) and hydrazine hydrate (0.05 mol) in EtOH (2.5 mL) were refluxed for 27 h. The progress of the reaction was monitored by TLC using CHCl_3_: MeOH (8:2) as the mobile phase. The 2/3rd volume of EtOH was removed under reduced pressure and the reaction mixture was then poured onto crushed ice. The resultant precipitate was filtered, washed with H_2_O, and dried. The crude product was recrystallized from EtOH. Yield: 56%; mp: 207 °C; ^1^H NMR (300 MHz, DMSO-*d*_6_) δ = 8.98 (s, 1H, CO-NH), 7.15 (s, 2H, SO_2_-NH_2_), 4.40 (s, 2H, NH_2_), 7.60-7.68 (m, 5H, Ar-NH and C–H aromatic); IR (KBr) cm^−1^: 3365 (N-H *str* of NH_2_), 3281 (N-H *str*), 3074 (aromatic C-H *str*), 1676 (C=O *str*), 1521 (aromatic C=C *str*), 1408, 1301, 1153 (S=O *str*).

#### 4.1.3. Synthesis of (E)-1-(4-substituted phenyl)-3-substituted Aromatic prop-2-en-1-one 4 [20,21]

Substituted acetophenone (0.01 mol) and various substituted aldehydes (0.01 mol) were mixed in ethanol (40 mL) in a conical flask placed in an ice bath. To this, 60% NaOH solution (10 mL) was added dropwise with continuous stirring for 30 min (in ice bath). The mixing was continued for another 2–3 h, maintaining the ice bath. The mixture was kept in a refrigerator overnight. Reaction completion was confirmed by TLC (hexane:ethyl acetate = 2:1). Then, it was diluted with ice-cold water, filtered, washed well with cold water, dried in air, and recrystallized from rectified methanol.

#### 4.1.4. General Procedure for Synthesis of 3-phenyl-5-aryl-*N*-(4-sulfamoylphenyl)-4,5-dihydro-1*H*-pyrazole-1-carboxamide 5–25 [22]

A mixture of chalcones (*E*)-1-(substituted phenyl)-3-substituted aromatic prop-2-en-1-one **4** (0.01 mol) and 4-aminosulfonylphenyl semicarbazide **3** (0.02 mol) in 20 mL ethanol was refluxed for 2 h with stirring (heating mantle was used with a temperature of 70–80 °C). To this boiling mixture, alcoholic KOH solution (10 mL, 60%) was added dropwise with continuous stirring for 30 min (with refluxing at the same temperature: 70–80 °C). This mixture was refluxed further for 2 h and then kept for overnight stirring at room temperature. The reaction was monitored by TLC using methanol/chloroform (20:80). The resulting solution was poured on slush and the precipitate was filtered and recrystallized from ethanol. Compounds **5**–**25** were synthesized according to the above general procedure.

##### 3,5-Diphenyl-4,5-dihydro-pyrazole-1-carboxylic acid (4-sulfamoyl-phenyl)-amide **5**

Yield = 60%; m.p. 242 °C; Purity (HPLC): 95.2%; ^1^H NMR (300 MHz, DMSO *d*_6_) δ = 9.46 (s, 1H, Ar-NH), 7.98–7.24 (m, 13H, Ar-H), 7.20 (_S_, 2H, SO_2_-NH_2_), 3.23–3.16 (dd, 1H, CHH_a_), 3.96–3.48 (dd, 1H, CHH_b_), 5.58–5.53 (dd, 1H, CH); ^13^C NMR (75 MHz, DMSO-*d*_6_): δ = 152.81, 151.48, 143.23, 142.93, 142.83, 137.67, 131.51, 130.53, 128.97, 128.32, 127.69, 127.36, 126.9, 118.83, 60.63, 42.83; Mass *m*/*z*: 421 (M + 1)^+^; IR max (KBr): 3336, 3375 (NH *str* of NH_2_), 3279, (NH *str*), 3063(Ar C-H*str*), 1674 (C=O *str*), 1585 (C=N *str*). 

##### 5-(4-Chlorophenyl)-3-phenyl-4,5-dihydro-pyrazole-1-carboxylic acid (4-sulfamoyl-phenyl)-amide **6**

Yield = 63%; m.p. 248 °C; Purity (HPLC): 88.55%; ^1^H NMR (300 MHz, DMSO *d*_6_) δ = 9.45 (s, 1H, Ar-NH), 7.98–7.14 (m, 12H, Ar-H), 7.20 (s, 2H, SO_2_-NH_2_), 3.29–3.17 (dd, 1H, CHH_a_), 3.95–3.84 (dd, 1H, CHH_b_), 5.60–5.54 (dd, 1H, CH); ^13^C NMR (75 MHz, DMSO-*d*_6_): δ = 153.13, 151.43, 142.75, 142.08, 140.61, 137.07, 132.43, 129.94, 129.03, 128.33, 128.01, 127.39, 126.89, 119.0, 60.09, 42.6; Mass *m*/*z*: 455 (M + 1)^+^; IR max (KBr): 3365 (NH *str* of NH_2_), 3232, (NH *str*), 3063 (Ar C-H*str*), 1685 (C=O *str*), 1587 (C=N *str*), 790 (C-Cl).

##### 5-(4-Flurophenyl)-3-phenyl-4,5-dihydro-pyrazole-1-carboxylic acid (4-sulfamoyl-phenyl)-amide **7**

Yield = 58%; m.p. 246 °C; Purity (HPLC): 100%; ^1^H NMR (300 MHz, DMSO *d*_6_) δ = 9.46 (s, 1H, Ar-NH), 7.97–7.27 (m, 12H, Ar-H), 7.20 (s, 2H, SO_2_-NH_2_), 3.25–3.17 (dd, 1H, CHH_a_), 3.95–3.85 (dd, 1H, CHH_b_), 5.59–5.53 (dd, 1H, CH); ^13^C NMR (75 MHz, DMSO-*d*_6_): δ = 160.61, 152.86, 151.42, 142.78, 134.32, 131.48, 130.57, 128.98, 128.18, 128.1, 127.39, 126.87, 118.87, 115.91, 60.01, 42.7; Mass *m*/*z*: 439 (M + 1)^+^; IR max (KBr): 3335 (NH *str* of NH_2_), 3234, (NH *str*), 3059 (Ar C-H*str*), 1683 (C=O *str*), 1585 (C=N*str*), 1411 (Ar-F). 

##### 5-(2-Chloro-6-fluorophenyl)-3-phenyl-4, 5-dihydro-pyrazole-1-carboxylic acid (4-sulfamoyl-phenyl)-amide **8**

Yield = 58%, m.p. 244 °C, Purity (HPLC): 97.55%; ^1^H NMR (300 MHz, DMSO *d*_6_) δ = 9.41 (s, 1H, Ar-NH), 7.97–7.23 (m, 11H, Ar-H), 7.20 (s, 2H, SO_2_-NH_2_), 3.41–3.28 (dd, 1H, CHH_a_), 3.96–3.86 (dd, 1H, CHH_b_), 6.04–5.97 (dd, 1H, CH); ^13^C NMR (75 MHz, DMSO-*d*_6_): δ = 152.96, 152.87, 151.36, 142.88, 142.39, 137.74, 134.62, 130.53, 129.12, 127.39, 127.08, 126.88, 126.6, 119.04, 116.08; Mass *m*/*z*: 473 (M + 1)^+^;IR max (KBr): 3389, 3327 (NH *str* of NH_2_), 3240, (NH *str*), 3069 (Ar C-H*str*), 1685 (C=O *str*), 1585 (C=N *str*), 1410 (C-F), 790 (C-Cl).

##### 3-Phenyl-5-thiophene-2-yl-4, 5-dihydro-pyrazole-1-carboxylic acid (4-sulfamoyl-phenyl)-amide **9**

Yield = 56%; m.p. 249 °C; Purity (HPLC): 95.23%; ^1^H NMR (300 MHz, DMSO *d*_6_) δ = 9.44 (s, 1H, Ar-NH), 8.01–6.96 (m, 12H, Ar-H), 7.21 (s, 2H, SO_2_-NH_2_), 3.6–3.4 (dd, 1H, CHH_a_), 4.0–3.88 (dd, 1H, CHH_b_), 6.0–5.9 (dd, 1H, CH); ^13^C NMR (75 MHz, DMSO-*d*_6_): δ = 153.04, 151.44, 142.83, 142.58, 137.89, 129.02, 128.63, 128.08, 127.86, 127.17, 126.95, 119.07, 56.24, 42.53; Mass *m*/*z*: 427 (M + 1)^+^; IR max (KBr): 3362, 3327 (NH *str* of NH_2_), 3279, (NH *str*), 3063 (Ar C-H*str*), 1685 (C=O *str*), 1585 (C=N *str*), 711 (C-S). 

##### 5-Anthracene-9-yl-3-phenyl-4,5-dihydro-pyrazole-1-carboxylic acid (4-sulfamoyl-phenyl)-amide **10**

Yield = 58%; m.p. 252 °C; Purity (HPLC): 94.64%; ^1^H NMR (300 MHz, DMSO *d*_6_) δ = 9.56 (s, 1H, Ar-NH), 8.71–7.38 (m, 18H, Ar-H), 7.14 (s, 2H, SO_2_-NH_2_), 3.57–3.47 (dd, 1H, CHH_a_), 4.22–4.12 (dd, 1H, CHH_b_), 7.08–7.01 (dd, 1H, CHH_X_); ^13^C NMR (100 MHz, DMSO-*d*_6_): δ = 153.23, 152.18, 142.9, 140.96, 137.69, 132.01, 131.54, 130.75, 129.26, 128.39, 127.88, 127.51, 126.97, 126.42, 125.35, 125.21, 124.31, 118.73, 56.71, 42.03; IR max (KBr): 3358, 3294 (NH *str* of NH_2_), 3223, (NH *str*), 3080 (Ar C-H*str*), 1680 (C=O *str*), 1587 (C=N *str*).

##### 5-(2,4-Dichlorophenyl)-3-phenyl-4, 5-dihydro-pyrazole-1-carboxylic acid (4-sulfamoyl-phenyl)-amide **11**

Yield = 68%; m.p. 168–170 °C; Purity (HPLC): 100%; ^1^H NMR (300 MHz, DMSO *d*_6_) δ = 9.38 (s, 1H, Ar-NH), 7.97–7.32 (m, 12H, Ar-H), 7.16 (s, 2H, SO_2_-NH_2_), 3.24–3.18 (dd, 1H, CHH_a_), 3.95–3.88 (dd, 1H, CHH_b_), 5.75–5.70 (dd, 1H, CHH_X_); ^13^C NMR (75 MHz, DMSO-*d*_6_): 152.81, 151.48, 143.23, 142.93, 142.83, 137.67, 131.51, 130.53, 128.97, 128.32, 127.69, 127.36, 126.9, 118.83, 60.63, 42.83; IR (ATR) cm^−1^: 3396, 3315 (N-H *str* of NH_2_), 3240 (N-H *str*), 3070 (aromatic C-H*str*), 1685 (C=O *str*), 1589 (C=N *str*), 1518 (aromatic C=C *str*), 1491, 1340, 1313, 1234, 1153 (S=O *str*), 756 (C-S *str*), 825 (aromatic C-Cl *str*).

##### 3-(4-Chlorophenyl)-5-phenyl-*N*-(4-sulfamoylphenyl)-4,5-dihydro-1*H*-pyrazole-1-carboxamide **12**

Yield = 75%; m.p. 216–218 °C; Purity (HPLC): 98.9%; ^1^H NMR (300 MHz, DMSO-*d*_6_) δ = 9.46 (s, 1H, Ar-NH), 7.98–7.24 (m, 13H, Ar-H), 7.20 (_S_, 2H, SO_2_-NH_2_), 3.23–3.16 (dd, 1H, CHH_a_), 3.96–3.48 (dd, 1H, CHH_b_), 5.58–5.53 (dd, 1H, CH);^13^C NMR (75 MHz, DMSO-*d*_6_): 152.8, 151.4, 143.2, 142.9, 142.8, 137.6, 131.5, 130.5, 128.9, 128.3, 127.6, 127.3, 126.9, 118.8, 60.6, 42.8; MS (*m*/*z*): 455 (M + 1)^+^; IR (ATR) cm^−1^: 3406, 3306 (N-H *str* of NH_2_), 3234 (N-H *str*), 3166, 3066, 3032 (aromatic C-H*str*), 1684 (C=O *str*), 1589 (C=N *str*), 1518 (aromatic C=C *str*) 1415, 1340, 1155 (S=O *str*), 754 (C-S *str*), 698 (aromatic C-Cl *str*).

##### 3-(4-Chlorophenyl)-*N*-(4-sulfamoylphenyl)-5-(p-tolyl)-4,5-dihydro-1*H*-pyrazole-1-carboxamide **13**

Yield = 70%; m.p. 220–221 °C; ^1^H NMR (300 MHz, DMSO *d*_6_) δ = 9.44 (s, 1H, Ar-NH), 7.97–7.19 (m, 12H, Ar-H), 7.14 (s, 2H, SO_2_-NH_2_), 2.34 (s, 3H, C-H of alkane), 3.26–3.13 (dd, 1H, CHH_a_), 3.93–3.83 (dd, 1H, CHH_b_), 5.54–5.48 (dd, 1H, CH); ^13^C NMR (75 MHz, DMSO-*d*_6_): 152.81, 151.48, 143.23, 142.93, 142.83, 137.67, 131.51, 130.53, 128.97, 128.32, 127.69, 127.36, 126.9, 118.83, 60.63, 42.83; MS (*m*/*z*): 469 (M + 1)^+^; IR (ATR) cm^−1^: 3342 (N-H *str* of NH_2_), 3250 (N-H *str*), 3198, 3082, 2926 (aromatic C-H*str*), 1680 (C=O *str*), 1585 (C=N *str*), 1519 (aromatic C=C *str*), 1417, 1317, 1234, 1151 (S=O *str*), 736 (C-S *str*), 815 (aromatic C-Cl *str*).

##### 3,5-Bis(4-chlorophenyl)-N-(4-sulfamoylphenyl)-4,5-dihydro-1H-pyrazole-1-carboxamide **14**

Yield = 68%; m.p. 182–183 °C; Purity (HPLC): 96.17%; ^1^H NMR (300 MHz, DMSO *d*_6_) δ = 9.45 (s, 1H, Ar-NH), 7.98–7.14 (m, 12H, Ar-H), 7.20 (s, 2H, SO_2_-NH_2_), 3.29–3.17 (dd, 1H, CHH_a_), 3.95–3.84 (dd, 1H, CHH_b_), 5.60–5.54 (dd, 1H, CH); ^13^C NMR (75 MHz, DMSO-*d*_6_): 152.81, 151.48, 143.23, 142.93, 142.83, 137.67, 131.51, 130.53, 128.97, 128.32, 127.69, 127.36, 126.9, 118.83, 60.63, 42.83; MS (*m*/*z*): 489 (M)^+^; IR (ATR) cm^−1^: 3354 (N-H *str* of NH_2_), 3259 (N-H *str*), 3097, 2926 (aromatic C-H*str*), 1689 (C=O *str*), 1587 (C=N *str*), 1525 (aromatic C=C *str*), 1406, 1315, 1236, 1136 (S=O *str*), 732 (C-S *str*), 817 (aromatic C-Cl *str*).

##### 3-(4-Chlorophenyl)-5-(4-fluorophenyl)-N-(4-sulfamoylphenyl)-4,5-dihydro-1H-pyrazole-1-carboxamide **15**

Yield = 72%; m.p. 198–200 °C; ^1^H NMR (300 MHz, DMSO *d*_6_) δ = 9.46 (s, 1H, Ar-NH), 7.97–7.27 (m, 12H, Ar-H), 7.20 (s, 2H, SO_2_-NH_2_), 3.25–3.17 (dd, 1H, CHH_a_), 3.95–3.85 (dd, 1H, CHH_b_), 5.59–5.53 (dd, 1H, CH); ^13^C NMR (75 MHz, DMSO-*d*_6_): 152.81, 151.48, 143.23, 142.93, 142.83, 137.67, 131.51, 130.53, 128.97, 128.32, 127.69, 127.36, 126.9, 118.83, 60.63, 42.83; MS (*m*/*z*): 473 (M + 1)^+^; IR (ATR) cm^−1^: 3390, 3306 (N-H *str* of NH_2_), 3234 (N-H *str*), 3074, 2939 (aromatic C-H*str*), 1683 (C=O *str*), 1589 (C=N *str*), 1518 (aromatic C=C *str*), 1413, 1340, 1313, 1219, 1155 (S=O *str*), 738 (C-S *str*), 817 (aromatic C-Cl *str*), 1087 (C-F *str*). 

##### 5-(2-Chlorophenyl)-3-(4-chlorophenyl)-N-(4-sulfamoylphenyl)-4,5-dihydro-1H-pyrazole-1-carboxamide **16**

Yield = 65%; m.p. 160–161 °C; Purity (HPLC): 100%; ^1^H NMR (300 MHz, DMSO *d*_6_) δ = 9.54 (s, 1H, Ar-NH), 7.97–7.32 (m, 12H, Ar-H), 7.21 (s, 2H, SO_2_-NH_2_), 3.27–3.10 (dd, 1H, CHH_a_), 4.04–3.94 (dd, 1H, CHH_b_), 5.8–5.78 (dd, 1H, CH); ^13^C NMR (75 MHz, DMSO-*d*_6_): 152.81, 151.48, 143.23, 142.93, 142.83, 137.67, 131.51, 130.53, 128.97, 128.32, 127.69, 127.36, 126.9, 118.83, 60.63, 42.83; MS (*m*/*z*): 489 (M)^+^; IR (ATR) cm^−1^: 3335 (N-H *str* of NH_2_), 3250 (N-H *str*), 3078, 2935 (aromatic C-H *str*), 1664 (C=O *str*), 1587 (C=N *str*), 1533 (aromatic C=C *str*), 1421, 1317, 1236, 1149 (S=O *str*), 734 (C-S *str*), 833 (aromatic C-Cl *str*).

##### 3-(4-Chlorophenyl)-5-(2,4-dichlorophenyl)-*N*-(4-sulfamoylphenyl)-4,5-dihydro-1*H*-pyrazole-1-carboxamide **17**

Yield = 70%; m.p. 180–182 °C; Purity (HPLC): 97.26%; ^1^H NMR (300 MHz, DMSO *d*_6_) δ = 9.53 (s, 1H, Ar-NH), 7.96–7.30 (m, 12H, Ar-H), 7.21 (s, 2H, SO_2_-NH_2_), 3.25–3.12 (dd, 1H, CHH_a_), 4.03–3.46 (dd, 1H, CHH_b_), 5.81–5.75 (dd, 1H, CH); ^13^C NMR (75 MHz, DMSO-*d*_6_): 152.81, 151.48, 143.23, 142.93, 142.83, 137.67, 131.51, 130.53, 128.97, 128.32, 127.69, 127.36, 126.9, 118.83, 60.63, 42.83; IR (ATR) cm^-1^: 3336 (N-H *str* of NH_2_), 3248 (N-H *str*), 3086, 2928 (aromatic C-H *str*), 1687 (C=O *str*), 1589 (C=N *str*), 1521 (aromatic C=C *str*), 1404, 1317, 1238, 1153 (S=O *str*), 732 (C-S *str*), 817 (aromatic C-Cl *str*).

##### 3-(4-Chlorophenyl)-N-(4-sulfamoylphenyl)-5-(thiophen-2-yl)-4,5-dihydro-1H-pyrazole-1-carboxamide **18**

Yield = 75%; m.p. 174–175 °C; Purity (HPLC): 93.71%; ^1^H NMR (300 MHz, DMSO *d*_6_) δ = 9.44 (s, 1H, Ar-NH), 8.01–6.96 (m, 12H, Ar-H), 7.21 (s, 2H, SO_2_-NH_2_), 3.43–3.27 (dd, 1H, CHH_a_), 3.86–3.88 (dd, 1H, CHH_b_), 5.90–5.85 (dd, 1H, CH); ^13^C NMR (75 MHz, DMSO-*d*_6_): 152.81, 151.48, 143.23, 142.93, 142.83, 137.67, 131.51, 130.53, 128.97, 128.32, 127.69, 127.36, 126.9, 118.83, 60.63, 42.83; MS (*m*/*z*): 461 (M + 1)^+^; IR (ATR) cm^−1^: 3352-3342 (N-H *str* of NH_2_), 3223 (N-H *str*), 3101, 3014, 2914 (aromatic C-H*str*), 1678 (C=O *str*), 1587 (C=N *str*), 1514 (aromatic C=C *str*), 1404, 1315, 1238, 1153 (S=O *str*), 689 (C-S *str*), 825 (aromatic C-Cl *str*). 

##### 5-(2-Chloro-6-fluorophenyl)-3-(4-chlorophenyl)-N-(4-sulfamoylphenyl)-4,5-dihydro-1H-pyrazole-1-carboxamide **19**

Yield = 70%; m.p. 182–184 °C; Purity (HPLC): 100%; ^1^H NMR (300 MHz, DMSO *d*_6_) δ = 9.41 (s, 1H, Ar-NH), 7.97–7.23 (m, 11H, Ar-H), 7.20 (s, 2H, SO_2_-NH_2_), 3.41–3.28 (dd, 1H, CHH_a_), 3.96–3.86 (dd, 1H, CHH_b_), 6.04–5.97 (dd, 1H, CH); ^13^C NMR (75 MHz, DMSO-*d*_6_): 152.81, 151.48, 143.23, 142.93, 142.83, 137.67, 131.51, 130.53, 128.97, 128.32, 127.69, 127.36, 126.9, 118.83, 60.63, 42.83; MS (*m*/*z*): 507 (M)^+^; IR (ATR) cm^-1^: 3387, 3352 (N-H *str* of NH_2_), 3252 (N-H *str*), 3084, 2962 (aromatic C-H*str*), 1678 (C=O *str*), 1587 (C=N *str*), 1525 (aromatic C=C *str*), 1456, 1406, 1317, 1238, 1149 (S=O *str*), 738 (C-S *str*), 827 (Aromatic C-Cl *str*), 1087 (C-F *str*).

##### 3-(4-Chlorophenyl)-5-(2-hydroxyphenyl)-N-(4-sulfamoylphenyl)-4,5-dihydro-1H-pyrazole-1-carboxamide **20**

Yield = 60%; m.p. 182–184 °C; Purity (HPLC): 100%; ^1^H NMR (300 MHz, DMSO *d*_6_) δ = 10.02 (s, 1H, ArOH), 9.21 (s, 1H, Ar-NH), 7.97–7.45 (m, 12H, Ar-H), 7.23 (s, 2H, SO_2_-NH_2_), 3.24–3.06 (dd, 1H, CHH_a_), 3.96–3.72 (dd, 1H, CHH_b_), 5.66–5.45 (dd, 1H, CH); ^13^C NMR (75 MHz, DMSO-*d*_6_): 152.81, 151.48, 143.23, 142.93, 142.83, 137.67, 131.51, 130.53, 128.97, 128.32, 127.69, 127.36, 126.9, 118.83, 60.63, 42.83; IR (ATR) cm^−1^:3217 (O-H str), 3352 (N-H *str* of NH_2_), 3282 (N-H *str*), 2993 (aromatic C-H*str*), 1680 (C=O *str*), 1585 (C=N *str*), 1531 (aromatic C=C *str*), 1460, 1406, 1311 (S=O *str*), 1236 (aromatic C–OH*str*), 754 (C-S *str*), 825 (Aromatic C-Cl *str*).

##### 3-(4-Chlorophenyl)-5-(4-methoxyphenyl)-*N*-(4-sulfamoylphenyl)-4,5-dihydro-1*H*-pyrazole-1-carboxamide **21**

Yield = 80%; m.p. 188–190 °C; Purity (HPLC): 100%; ^1^H NMR (300 MHz, DMSO *d*_6_) δ = 9.42 (s, 1H, Ar-NH), 7.97–6.88 (m, 12H, Ar-H), 7.20 (s, 2H, SO_2_-NH_2_), 3.30–3.14 (dd, 1H, CHH_a_), 3.91–3.80 (dd, 1H, CHH_b_),5.32–5.47 (dd, 1H, CH)3.71 (s, 3H, OCH3); ^13^C NMR (75 MHz, DMSO-*d*_6_): 152.81, 151.48, 143.23, 142.93, 142.83, 137.67, 131.51, 130.53, 128.97, 128.32, 127.69, 127.36, 126.9, 118.83, 60.63, 42.83; IR (ATR) cm^−1^:3358, 3315 (N-H *str* of NH_2_), 3205 (N-H *str*), 3099, 2966, 2837 (aromatic C-H*str*), 1670 (C=O *str*), 1587 (C=N *str*), 1514 (aromatic C=C *str*), 1406, 1319, 1242, 1155 (S=O *str*), 727 (C-S *str*), 825 (aromatic C-Cl *str*).

##### 3-(4-Chlorophenyl)-5-(3,4-dimethoxyphenyl)-*N*-(4-sulfamoylphenyl)-4,5-dihydro-1*H*-pyrazole-1-carboxamide **22**

Yield = 62%; m.p. 210–211 °C; Purity (HPLC): 81.93%; ^1^H NMR (300 MHz, DMSO *d*_6_) δ = 9.44 (s, 1H, Ar-NH), 7.97–6.72 (m, 11H, Ar-H), 7.20 (s, 2H, SO_2_-NH_2_), 3.29–3.16 (dd, 1H, CHH_a_), 3.91–3.78 (dd, 1H, CHH_b_), 5.52–5.46 (dd, 1H, CH) 3.72, 3.71 (s, 6H, 2OCH3); ^13^C NMR (75 MHz, DMSO-*d*_6_): 152.81, 151.48, 143.23, 142.93, 142.83, 137.67, 131.51, 130.53, 128.97, 128.32, 127.69, 127.36, 126.9, 118.83, 60.63, 42.83; IR (ATR) cm^−1^: 3352 (N-H *str* of NH_2_), 3254 (N-H *str*), 3093, 3009, 2941, 2837 (aromatic C-H *str*), 1681 (C=O *str*), 1587 (C=N *str*), 1510 (aromatic C=C *str*), 1419, 1315, 1261, 1153 (S=O *str*), 736 (C-S *str*), 802 (aromatic C-Cl *str*).

##### 3-(4-Bromophenyl)-5-phenyl-45-dihydro-pyrazole-1-carboxylic acid (4-sulfamoyl-phenyl)-amide **23**

Yield = 60%; m.p. 243 °C; ^1^H NMR (300 MHz, DMSO *d*_6_) δ = 9.4–9.6 (s, 1H, Ar-NH), 7.98–7.24 (m, 13H, Ar-H), 7.20 (_S_, 2H, SO_2_-NH_2_), 3.23–3.16 (dd, 1H, CHH_a_), 3.96–3.8 (dd, 1H, CHH_b_), 5.6–5.53 (dd, 1H, CH); IR max (KBr): 3404, 3363 (NH *str* of NH_2_), 3232, (NH *str*), 3091 (Ar C-H*str*), 1683 (C=O *str*), 1587 (C=N *str*).

##### 3-(4-Bromophenyl)-5-(4-chloro phenyl)-4,5-dihydro-pyrazole-1-carboxylic acid (4-sulfamoyl-phenyl)-amide **24**

Yield = 62%; m.p. 247 °C; Purity (HPLC): 96.38%; ^1^H NMR (300 MHz, DMSO *d*_6_) δ = 9.45 (s, 1H, Ar-NH), 7.98–7.14 (m, 12H, Ar-H), 7.20 (s, 2H, SO_2_-NH_2_), 3.29–3.17 (dd, 1H, CHH_a_), 3.95–3.84 (dd, 1H, CHH_b_), 5.60–5.54 (dd, 1H, CH); ^13^C NMR (100 MHz, DMSO-*d*_6_): δ = 151.8, 151.42, 142.74, 141.83, 140.61, 137.77, 132.55, 130.6, 129.18, 128.96, 127.92, 126.87, 118.91, 60.29, 42.46; IR max (KBr): 3362, 3300 (NH *str* of NH_2_), 3230, (NH *str*), 3088 (Ar C-H*str*), 1683 (C=O *str*), 1585 (C=N *str*).

##### 3-(4-Bromo phenyl)-5-(4-fluro phenyl)-4, 5-dihydro-pyrazole-1-carboxylic acid (4-sulfamoyl-phenyl)-amide **25**

Yield = 59%; m.p. 245 °C; ^1^H NMR (300 MHz, DMSO *d*_6_) δ = 9.46 (s, 1H, Ar-NH), 7.97–7.27 (m, 12H, Ar-H), 7.20 (s, 2H, SO_2_-NH_2_), 3.25–3.17 (dd, 1H, CHH_a_), 3.95–3.85 (dd, 1H, CHH_b_), 5.59–5.53 (dd, 1H, CH); ^13^C NMR (75 MHz, DMSO-*d*_6_): δ = 160.64, 153.29, 151.81, 143.5, 142.76, 137.85, 136.82, 129.64, 129.18, 128.71, 128.11, 126.85, 118.99, 115.79, 60.22, 42.55; IR max (KBr): 3362, 3294 (NH *str* of NH_2_), 3232, (NH *str*), 3082 (Ar C-H*str*), 1680 (C=O *str*), 1587 (C=N *str*), 1411 (C-F).

### 4.2. CA Inhibition

An Applied Photophysics stopped-flow instrument was used for assaying the CA catalyzed CO_2_ hydration activity [18]. Phenol red (at a concentration of 0.2 mM) was used as an indicator, working at the absorbance maximum of 557 nm, with 20 mM Hepes (pH 7.5) as a buffer, and 20 mM Na_2_SO_4_ (for maintaining constant the ionic strength), following the initial rates of the CA-catalyzed CO_2_ hydration reaction for a period of 10–100 s. The CO_2_ concentrations ranged from 1.7 to 17 mM for the determination of the kinetic parameters and inhibition constants. For each inhibitor, at least six traces of the initial 5%–10% of the reaction were used for determining the initial velocity. The uncatalyzed rates were determined in the same manner and subtracted from the total observed rates. Stock solutions of inhibitor (0.1 mM) were prepared in distilled-deionized water and dilutions up to 0.01 nM were performed thereafter with the assay buffer. Inhibitor and enzyme solutions were preincubated together for 15 min at room temperature prior to assay, in order to allow for the formation of the E–I complex. The inhibition constants were obtained by non-linear least-squares methods using PRISM 3 and the Cheng–Prusoff equation, as reported earlier [23,24], and represent the mean from at least three different determinations. All CA isoforms were recombinant ones obtained in-house as reported earlier [25,26,27].

### 4.3. Molecular Docking

The crystal structures of CA IX (pdb 5FL4) [19] and XII (pdb 1JD0) [8] were prepared using the Protein Preparation Wizard tool implemented in Maestro-Schrödinger suite, assigning bond orders, adding hydrogens, deleting water molecules, and optimizing H-bonding networks [28]. An energy minimization protocol with a root-mean-square deviation (RMSD) value of 0.30 was applied using an Optimized Potentials for Liquid Simulation (OPLS3e) force field. Three-dimensional ligand structures were prepared by Maestro [28] and evaluated for their ionization states at pH 7.4 ± 0.5 with Epik [28]. The OPLS3e force field in Macromodel [28] was used for energy minimization for a maximum number of 2500 conjugate gradient iterations and setting a convergence criterion of 0.05 kcal mol^−1^Å^−1^. The docking grid was centered on the center of mass of the co-crystallized ligands and Glide was used with default settings. Ligands were docked with the standard precision mode (SP) of Glide [28] and the best 5 poses of each molecule were retained as output. The best pose for each compound, evaluated in terms of coordination, hydrogen bond interactions, and hydrophobic contacts, was refined with Prime [28] with a VSGB solvation model considering the target flexible within 3 Å around the ligand [29,30,31].

## Figures and Tables

**Table 1:**
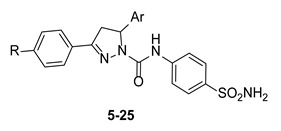
Inhibition data of human carbonic anhydrase (CA) isoforms I, II, VII, IX, and XII with sulfonamide derivatives **5**–**25** reported here and the standard sulfonamide inhibitor acetazolamide (AAZ) by a stopped-flow CO_2_ hydrase assay.

Cmpd	R	Ar	K_I_ (nM)				
			CA I	CA II	CA VII	CA IX	CA XII
**5**	H	C_6_H_5_	382	67.3	33.2	1.3	7.7
**6**	H	4-Cl-C_6_H_4_	36.2	8.2	7.7	1.4	1.2
**7**	H	4-F-C_6_H_4_	252	58.2	2.3	1.5	6.3
**8**	H	2-Cl-6-F-C_6_H_3_	38.3	5.2	2.4	1.5	1.4
**9**	H	thiophen-2-yl	185	5.2	1.9	1.4	2.5
**10**	H	anthracene-9-yl	293	7.1	2.4	1.3	6.8
**11**	H	2,4-diCl-C_6_H_3_	39.5	5.4	2.5	1.4	0.84
**12**	Cl	C_6_H_5_	239	233	350	16.5	9.2
**13**	Cl	4-CH_3_-C_6_H_4_	66.7	12.3	4.5	2.9	9.2
**14**	Cl	4-Cl-C_6_H_4_	237.9	12.7	3.3	11.4	1.1
**15**	Cl	4-F-C_6_H_4_	616.7	71.3	3.2	2.7	1.0
**16**	Cl	2-Cl-C_6_H_4_	296.6	8.8	4.3	8	1.7
**17**	Cl	2,4-diCl-C_6_H_3_	769.6	13.5	27.6	15	1.3
**18**	Cl	thiophen-2-yl	726	9.2	2.9	2.5	0.62
**19**	Cl	2-Cl-6-F-C_6_H_3_	42.7	10.8	2.3	2.7	6.8
**20**	Cl	2-OH-C_6_H_4_	74.2	7.6	2.1	76.6	0.99
**21**	Cl	4-OCH_3_-C_6_H_4_	367.7	9.4	2.7	2.8	0.82
**22**	Cl	3,4-diOCH_3_-C_6_H_3_	165.9	9.3	2.3	2.2	1.5
**23**	Br	C_6_H_5_	44.4	6.3	2.9	1.4	6.7
**24**	Br	4-Cl-C_6_H_4_	45.5	10.7	2.7	14.3	0.89
**25**	Br	4-F-C_6_H_4_	38.2	5.5	2.6	1.4	2.4
**AAZ**	-		250	12.1	2.5	25	5.8

**Table 2:** Docking score (Glide) of the predicted poses of *(R)*- and *(S)*-enantiomers of **5–25** and AAZ bound to CA IX (pdb 5FL4) and CA XII (pdb 1JD0).

Cmpd	GlideScore (kcal/mol)		Cmpd	GlideScore (kcal/mol)	
	CA IX	CA XII		CA IX	CA XII
***(S)*-5**	−6.82	−6.02	***(R)*-5**	−5.34	−6.84
***(S)*-6**	−6.54	−6.28	***(R)*-6**	−5.67	−7.01
***(S)*-7**	−6.93	−5.63	***(R)*-7**	−5.39	−6.49
***(S)*-8**	−6.28	−6.45	***(R)*-8**	−5.38	−7.36
***(S)*-9**	−6.34	−6.63	***(R)*-9**	−5.94	−7.57
***(S)*-10**	−7.01	−6.22	***(R)*-10**	−5.68	−6.82
***(S)*-11**	−6.35	−5.96	***(R)*-11**	−5.02	−7.69
***(S)*-12**	−5.86	−6.29	***(R)*-12**	−5.49	−6.57
***(S)*-13**	−6.57	−6.07	***(R)*-13**	−5.79	−7.03
***(S)*-14**	−6.02	−6.38	***(R)*-14**	−5.38	−7.51
***(S)*-15**	−6.83	−5.99	***(R)*-15**	−5.46	−7.36
***(S)*-16**	−6.12	−6.23	***(R)*-16**	−5.82	−7.15
***(S)*- ** **17**	−5.84	−6.54	***(R)*- ** **17**	−5.09	−7.64
***(S)*- ** **18**	−6.14	−6.08	***(R)*- ** **18**	−5.97	−7.92
***(S)*- ** **19**	−6.72	−6.63	***(R)*- ** **19**	−5.01	−7.29
***(S)*- ** **20**	−5.67	−6.41	***(R)*- ** **20**	−5.26	−6.86
***(S)*- ** **21**	−6.35	−6.39	***(R)*- ** **21**	−6.03	−7.27
***(S)*- ** **22**	−6.92	−6.28	***(R)*- ** **22**	−6.05	−7.16
***(S)*- ** **23**	−6.54	−6.92	***(R)*- ** **23**	−5.65	−6.48
***(S)*- ** **24**	−6.24	−6.47	***(R)*- ** **24**	−5.24	−7.58
***(S)*- ** **25**	−6.82	−6.19	***(R)*- ** **25**	−5.37	−7.22
**AAZ**	−6.31	−7.35			
